# A Vondrak Low Pass Filter for IMU Sensor Initial Alignment on a Disturbed Base

**DOI:** 10.3390/s141223803

**Published:** 2014-12-10

**Authors:** Zengke Li, Jian Wang, Jingxiang Gao, Binghao Li, Feng Zhou

**Affiliations:** 1 School of Environment and Spatial Informatics, China University of Mining and Technology, Xuzhou 22116, China; E-Mails: zengkeli@cumt.edu.cn (Z.L.); wjiancumt@yeah.net (J.W.); 2 School of Surveying and Spatial Information Systems, The University of New South Wales, Sydney 2052, Australia; E-Mail: binghao.li@unsw.edu.au; 3 School of Information Science Technology, East China Normal University, Shanghai 200062, China; E-Mail: zhouforme@gmail.com

**Keywords:** initial alignment, Vondrak filter, genetic algorithms, disturbed base

## Abstract

The initial alignment of the Inertial Measurement Unit (IMU) is an important process of INS to determine the coordinate transformation matrix which is used in the integration of Global Positioning Systems (GPS) with Inertial Navigation Systems (INS). In this paper a novel alignment method for a disturbed base, such as a vehicle disturbed by wind outdoors, implemented with the aid of a Vondrak low pass filter, is proposed. The basic principle of initial alignment including coarse alignment and fine alignment is introduced first. The spectral analysis is processed to compare the differences between the characteristic error of INS force observation on a stationary base and on disturbed bases. In order to reduce the high frequency noise in the force observation more accurately and more easily, a Vondrak low pass filter is constructed based on the spectral analysis result. The genetic algorithms method is introduced to choose the smoothing factor in the Vondrak filter and the corresponding objective condition is built. The architecture of the proposed alignment method with the Vondrak low pass filter is shown. Furthermore, simulated experiments and actual experiments were performed to validate the new algorithm. The results indicate that, compared with the conventional alignment method, the Vondrak filter could eliminate the high frequency noise in the force observation and the proposed alignment method could improve the attitude accuracy. At the same time, only one parameter needs to be set, which makes the proposed method easier to implement than other low-pass filter methods.

## 1. Introduction

In the integration between GPS and INS, GPS has long-term stability, and there is no error accumulation over time, while the INS is completely autonomous, and generally has a high update rate with the attitude output [1]. The integrated navigation system can integrate these advantages and overcome the individual drawbacks, and can be used for providing various navigation information elements (position, velocity and attitude) [2].The initial alignment work is an INS process and the first step to be resolved for GPS/INS integrated system. The alignment of the strapdown inertial navigation system (SINS) is an important process to determine the coordinate transformation matrix between a navigation frame and a body frame before a navigation work begins [3]. At the same time, alignment accuracy has a key influence on estimation of the attitude, velocity and position of a vehicle. Good initial alignment performance will lead to high accuracy navigation.

The basic concept of INS alignment is quite simple and straightforward. If accurate navigation is to be achieved over long periods of time without auxiliary observation [4], accurate alignment is decisive. Since INS is entirely self-contained, it can align itself by using the measurements of local gravity and Earth rotation rate. Normally, the alignment process is divided into the coarse alignment and fine alignment [5]. The purpose of coarse alignment is to obtain appropriate attitude information which will be regarded as the initial value in the fine alignment process.

A high-accuracy INS can realize the alignment process by itself, while the low cost INS need the auxiliary conditions. Measurement noise in the INS is the key problem that influences the alignment accuracy. Some research to diminish the noise was proposed to achieve higher accuracy alignment performance. The characteristics of gyrocompass alignment errors were investigated from a stochastic theoretical point and the two kinds of covariance analysis approaches were presented [6]. This approach simplified the covariance analysis which made the initial error covariance matrix adopt a diagonal form. Noureldin proposed a new filtering approach that significantly reduced the angle random walk of the fiber optic gyroscope's output to a level that can ensure an accurate measurement of the Earth's rotation rate [7]. El-Sheimy suggested using multiple levels of wavelet decomposition to remove the high frequency noise components in gyro and accelerometer measurements [8]. The results showed that accurate alignment procedure and fast convergence of the estimation algorithm, in addition to reducing the estimation covariance of the three attitude angles, could be obtained. To reduce these alignment errors (lever arm effect, measurement time delay and ship-body flexure), an error compensation method based on state augmentation and robust state estimation was also devised [9]. The H ∞ filter was introduced to account for modeling uncertainties of time delay and the ship-body flexure. Junxiang proposed an inertial frame based alignment (IFBA) method [10], especially for the applications on a rocking platform. The IFBA method achieved the alignment by virtue of a cascade of low-pass FIR filters, which attenuated the disturbing acceleration and maintained the gravity vector. For rocket navigation systems, an algorithm for the initial alignment method was also investigated [11]. To deal with the large misalignments problem in initial alignment, nonlinear filtering methods are introduced. A novel scheme for Doppler Velocity Log aided INS alignment using an Unscented Kalman Filter is effective with any initial heading errors [12]. A Finite Impulse Response (FIR) filter was utilized to decrease the noise in accelerometers' measurements. In order to solve the problem of a IMU on a vibrating base, a ground fine alignment method is proposed [13] and the effects caused by the angular vibration are considered as system and measurement noise. Although these methods are able to diminish the noise in IMU observations to some extent, too many parameters need to be designed, which will lead to poor stability and complex calculations. For example, more than five key parameters are calculated in the cascade of low-pass FIR filters [10], besides the choice of filter type.

In the present study, an improved IMU initial alignment method using a Vondrak low pass filter is proposed to get more accurate attitude information for a disturbed base. The objective of this study is to realize high frequency noise elimination much more accurately and more easily and to improve the initial alignment accuracy for IMUs with disturbances. The paper is divided into seven sections. Following this introduction, the basic principles of initial alignment including coarse alignment and fine alignment are overviewed in Section 2. Section 3 describes the spectral analysis for IMU force observation to study the IMU error characteristics under the condition with disturbance. Based on the analysis result in Section 3, the Vondrak low pass filter aided by genetic algorithms for disturbance in force observation is proposed in Section 4. Section 5 reveals the improved initial alignment method proposed in the paper. Results of simulation experiments and field experiments are then presented and analyzed in Section 6, followed by a summary of the main conclusions.

## 2. Initial Alignment

### 2.1. Coarse Alignment

Coarse alignment algorithms use the force and angular rate observation with knowledge of the navigation frame gravity vector and Earth rotation rate vector to calculate the initial attitude [14]. The system is assumed to be stationary and the position latitude and longitude are known. The gravity vector and Earth rotation rate vector in the navigation frame can be written as:

(1) $$g^{n}={\left[\begin{array}{ccc} 0 & 0 & -g \end{array}\right]}^{T}$$

(2) $$\omega_{\text{ie}}^{n}={\left[\begin{array}{ccc} 0 & \omega_{\text{ie}}\cos L & \omega_{\text{ie}}\sin L \end{array}\right]}^{T}$$

where ω_ie_ = 0.000072921151467 rad/s is the Earth rotation rate, *g* is the local weight acceleration of gravity and *L* is the latitude.

The approximate value of *g* is:

(3) $$g=g_{0}\left(1+0.00527094\sin^{2}L+0.0000232718\sin^{4}L\right)-0.000003086h$$

where *g*_0_ = 9.7803267714*m*/*s*^2^ and *h* is the height.

Based on the given value ***g****^n^* and $\omega_{\text{ie}}^{n}$, the coarse alignment work can be achieved with the IMU force observation ***f****^b^* and angular rate observation $\omega_{\text{ie}}^{b}$.

In order to solve the all unknown elements in the orthogonal matrix, new vectors should be constructed for more equations. Assume that ξ and β are known in the navigation frame and measured in the body frame. The auxiliary vector is defined χ = ξ × β. The relation of the two sets of vectors can be shown as:

(4) $$\left[\begin{array}{ccc} \xi^{n} & \beta^{n} & \chi^{n} \end{array}\right]=C_{b}^{n}\;\left[\begin{array}{ccc} \xi^{b} & \beta^{b} & \chi^{b} \end{array}\right]$$

If ξ^n^ is the gravity vector, β^n^ is the Earth rotation rate vector, ξ^b^ is the force observation and β^b^ is the angular rate observation, then the direction cosine matrix $C_{n}^{b}$ can be calculated:

(5) $$C_{n}^{b}={\left(C_{b}^{n}\right)}^{-1}=\left[\begin{array}{ccc} f^{b} & \omega_{\text{ie}}^{b} & f^{b}{\times \omega }_{\text{ie}}^{b} \end{array}\right]\;\left[\begin{array}{ccc} \frac{1}{g}\tan L & 0 & \frac{1}{g}\\ \frac{1}{\omega_{\text{ie}}\cos L} & 0 & 0\\ 0 & \frac{1}{\omega_{\text{ie}}g\cos L} & 0 \end{array}\right]$$

Implementation of the above approach requires high quality gyros with sufficient precision and accuracy to measure the Earth rotation rate.

### 2.2. Fine Alignment

**Figure 1.**
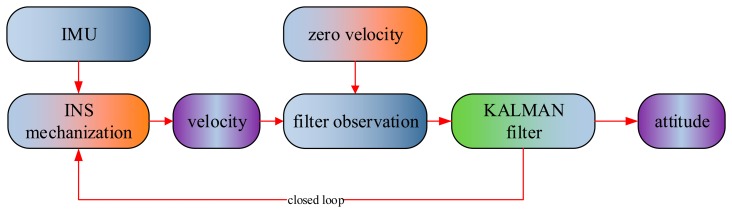
The fine alignment.

After the coarse alignment, an initial direction cosine matrix can be obtained. If more accurate attitude is required, the fine alignment will be processed. The fine alignment will depend on auxiliary information to get the attitude result with an optimal estimation method, such as a Kalman filter. The commonly used auxiliary information is that the velocity value in navigation frame is zero under static conditions. In the paper, the difference between the velocity calculated by INS mechanization and the zero velocity is regarded as the filter observation in the Kalman filter. Actually, the fine alignment is one process to modify the coarse alignment result of. Figure 1 shows the fine alignment algorithm flow.

The system error dynamic model of fine alignment used in the Kalman filter [15] is designed based on the IMU error equations. The insignificant terms are neglected in the linearization process. The psi-angle error equations of INS are as follows [1]:

(6) $$\delta \dot{r}=-\omega_{\text{en}}\times \delta r+\delta v$$

(7) $$\delta \dot{v}=-(2\omega_{\text{ie}}+\omega_{\text{en}})\times \delta v-\delta \psi \times f+\eta$$

(8) $$\delta \dot{\psi }=-(\omega_{\text{ie}}+\omega_{\text{en}})\times \delta \psi +ɛ$$

where δr, δv and δψ are the position, velocity and orientation error vectors, respectively. ω_en_ is the rate of navigation frame with respect to Earth, and ω_ie_ is the rate of Earth with respect to the inertial frame. The system error dynamics is obtained by expanding the accelerometer bias error vector η and the gyro drift error vector ɛ.

The accelerometer bias error vector η and the gyro drift error vector ɛ are regarded as the random walk process vectors, which are modeled as follows [16]:

9) $$\dot{\eta }=u_{\eta }$$

(10) $$\dot{ɛ}=u_{ɛ}$$

where u_η_ and u_ɛ_ are white Gaussian noise vectors.

By combining Equations (6) to (10), the system dynamical model becomes:

(11) $$\left\{ \begin{array}{l} \delta \dot{r}=-\omega_{\text{en}}\times \delta r+\delta v\\ \delta \dot{v}=-(\omega_{\text{ie}}+\omega_{\text{in}})\times \delta v-\delta \psi \times f+\eta \\ \delta \dot{\psi }=-\omega_{\text{in}}\times \delta \psi +ɛ\\ \dot{\eta }=u_{\eta }\\ \dot{ɛ}=u_{ɛ} \end{array}\right.$$

which can be generalized in matrix and vector form:

(12) $$\dot{X}=\Phi X+u$$

where in ***X*** is the error state vector, Φ is the system transition matrix, and ***u*** is the process noise vector.

The observation model in INS fine alignment is composed velocity difference vector between the real velocity and the INS computation value:

(13) $$\begin{array}{ll} Z_{v}(t) & =v_{R}(t)-v_{\text{INS}}(t)\\ & =v_{R}(t)-(v_{\text{INS}}(t-\Delta t)+\alpha (t)\cdot \Delta t) \end{array}$$

where ***Z****_v_*(*t*) is the velocity error measurement vector, ***v****_R_*(*t*) is the real velocity vector, ***v****_INS_*(*t*) is the INS velocity vector, *α*(*t*) is the acceleration vector determined by the INS alone, and Δ*t* is the sample time of INS. In the static condition, the real velocity vector ***v****_R_*(*t*) is the zero vector.

The generic observation equation system of the Kalman filter can be written as:

(14) $$Z_{k}=B_{k}X_{k}+\tau$$

where ***B****_k_* is the observation matrix, and τ is the measurement noise vector, assumed to be white Gaussian noise.

The optimal estimates of the state vector from the Kalman filter can be reached through a time update and a measurement update at a time instant:

(15) $${\hat{X}}_{k}={\hat{X}}_{k,k-1}+K_{k}(Z_{k}-B_{k}{\hat{X}}_{k,k-1})$$

(16) $$K_{k}=P_{k,k-1}B_{k}^{T}{\left(B_{k}P_{k,k-1}B_{k}^{T}+R_{k}\right)}^{-1}$$

(17) $${\hat{X}}_{k,k-1}=\Phi_{k,k-1}{\hat{X}}_{k-1}$$

(18) $$P_{k,k-1}=\Phi_{k}P_{k-1}\Phi_{k}^{T}+Q_{k}$$

(19) $$P_{k}=(I-K_{k}B_{k})P_{k,k-1}$$

where ***K****_k_* is the gain matrix of Kalman filter at *k* time, ***P****_k_* is the covariance matrix of state vector at *k* time, ***R****_k_* is the covariance matrix of measurement noise vector at *k* time, ***Q****_k_* is the covariance matrix of process noise at *k* time, and the subscript *k*, *k*−*1* represent the state and covariance estimates forward from *k*−*1* time to *k* time.

In the fine alignment, a feedback loop is used for correcting the systematic errors. In this way, the mechanization performs simple navigation calculation under the assumption of small errors. In this case, the error states will be reset to zero after every measurement update [17]. Thus, the navigation resolution is expressed by:

(20) $${\hat{X}}_{k}=P_{k,k-1}B_{k}^{T}{\left(B_{k}P_{k,k-1}B_{k}^{T}+R_{k}\right)}^{-1}Z_{k}$$

## 3. Spectral Analysis

The circumstances where the vehicle is located is one important influencing factor of alignment accuracy and speed. In good conditions, only coarse alignment will provide good performance. To the contrary, the Kalman algorithm is used to obtain more accurate attitude a long time after the coarse alignment in poor conditions. In order to analyze the frequency characteristics, the INS raw data in a marble checking platform indoors (condition one) and in a vehicle outdoors (condition two) were collected. The spectral analysis was processed based on procedures employing the multitaper method [18]. INS data (200 Hz) were received and stored. The INS in the vehicle will be disturbed by engine vibrations, getting on or off the bus, wind and so on.

**Figure 2.**
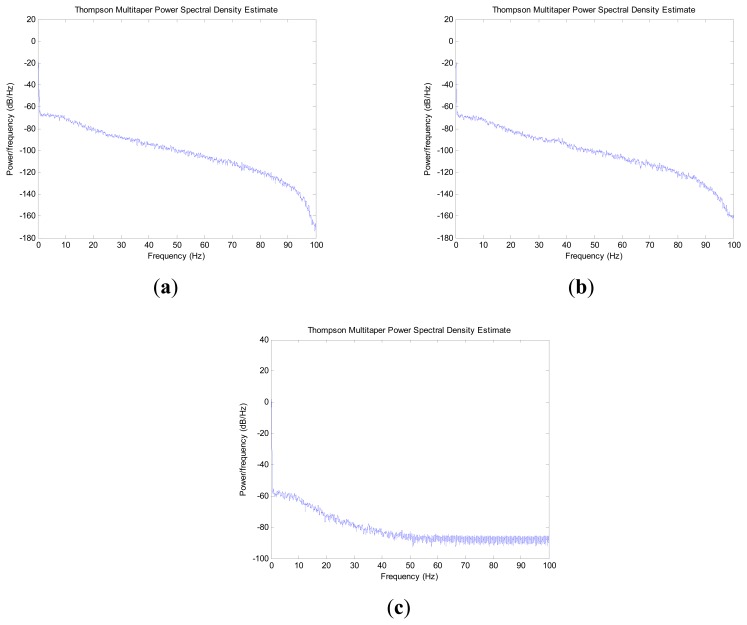
Spectral analysis of force observation in marble checking platform indoor: (**a**) in X direction; (**b**) in Y direction; (**c**) in Z direction.

**Figure 3.**
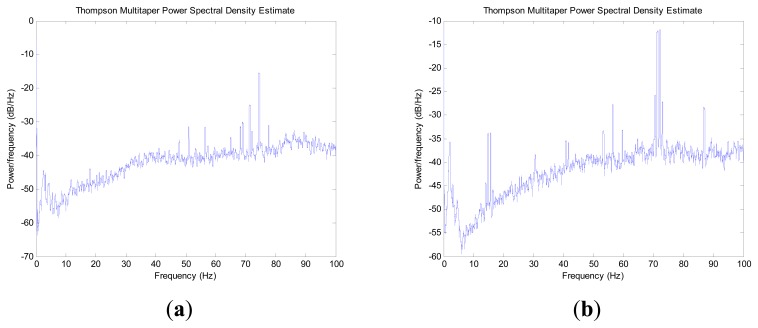

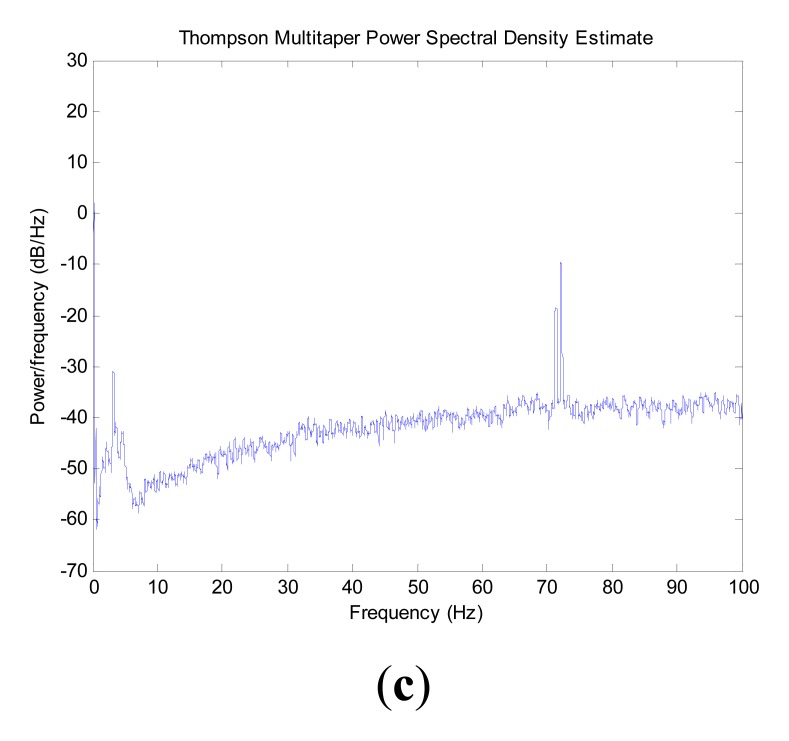
Spectral analysis of force observation in vehicle outdoor: (**a**) in X direction; (**b**) in Y direction; (**c**) in Z direction.

The power spectrum of force observations from the X, Y and Z directions in the inertial frame are shown in Figures 2 and 3. Comparing the above two conditions, the spectral analysis plot shows a similar result from 0 Hz to 0.5 Hz. From a frequency of more than 0.5 Hz to the end, the spectral analysis plot shows an approximately linear increase with a slope of 0.46 in condition one. At the same time, the spectral analysis plot shows no regularity in condition two. The force observations in condition two cover a much larger range of frequencies than that in condition one.

When the IMU sensor is located on the indoor marble checking platform, the power value of the frequency of less than 0.5 Hz is higher than that for more than 0.5 Hz. The power value decreases fast from 0.5 Hz to the end. When the INS sensor is located in the vehicle outdoor, the frequency power value has no regularity. In the whole frequency domain, lots of bulges appear. The result indicates that the frequency of disturbance for a disturbed IMU base is larger than 0.5 Hz. If one ideal low pass filter is designed, the high frequency part of force observation can be restrained and the low frequency part can be retained, which contributes to improving the alignment accuracy and shortening the convergence time.

The FIR filter and Vondrak filter are able to realize the low-pass filter work. In general, design of a cascade of FIR filters includes many parameters. To the contrary, the Vondrak filter only needs the cut-off frequency without the other parameters. The Vondrak filter thus has better adaptability and is chosen to process the force observations in this paper.

## 4. Vondrak Filter Aided Genetic Algorithms

### 4.1. Vondrak Filter

A sequence of measurement can be expressed as (*x_i_*, *y_i_*), *I* = 1, 2,…*N*, where *x_i_* and *y_i_* are the observation time and the observation value, respectively. The basic principle of the Vondrak filter is to compute the filter value according to that [19]:

(21) $$Q=F+\lambda^{2}S\to \min$$

with:

(22) $$F=\sum_{i=1}^{N}p_{i}{\left(y_{i}^{\prime }-y_{i}\right)}^{2}$$

(23) $$S=\sum_{i=1}^{N-3}{\left(\Delta^{3}y_{i}^{\prime }\right)}^{2}$$

where $y_{i}^{\prime }$ is the filter value corresponding to original observation value *y_i_*, *p_i_* is the weight of *y_i_*, $\Delta^{3}y_{i}^{\prime }$ is the third-difference of filter values calculated based on a cubic Lagrange polynomial and λ^2^ is a positive coefficient which regulates the relations between the degree of filtering and smoothness in the smoothing process.

In deriving the solution, a cubic Lagrange polynomial is fitted to four adjacent points (*x_i_*,*y′_i_*), (*x_i_*_+1_, *y′_i_*_+1_) (*x_i_*_+2_, *y′_i_*_+2_) and (*x_i_*_+3_, *y′_i_*_+3_) when considering points (*x_i_*_+1_, *y′_i_*_+1_) and (*x_i_*_+2_, *y′_i_*_+2_). A set of linear equations are formed based on Equation (21) to solve for the filter values.

When the coefficients λ^2^ tend to zero, *F* should tend to zero to get the minimum of Equation (21). The smooth values are close to the observation values. A rough curve will be derived and the operation is referred to as absolute fitting. When the coefficients λ^2^ tends to ∞, *S* should tend to zero to get the minimum of Equation (21) and a smooth cubic parabola will be obtained, which is called the absolute smoothing operation. Here, θ = 1/λ^2^ is called the smoothing factor which determines the degree of smoothing of the filtering curve. In general, the smaller the value θ, the stronger the degree of smoothing. Otherwise, the degree of smoothing is weak. In the Vondrak filter, the smoothing factor is the only design parameter that needs to be computed.

In fact, this method is a kind of joint distribution, which is constructed by a Gaussian distribution of measurement and the prior distribution of the third-difference of it. Compared with other smoothing methods, the major advantages of the method are that no predefined fitting function is derived, and that the filter values at the two ends of the time series can be calculated easily.

The Vondrak filter is applicable to equal-interval and unequal-interval observations. It can also be used to smooth time series and as a digital filter to wipe out high frequency information. The frequency response function of Vondrak filter can be written as [20]:

(24) $$G\left(\theta ,f\right)=\frac{1}{1+\theta^{-1}{\;(2\pi f)}^{6}}$$

where *f* is the frequency value.

If the exact smoothing factor is selected, the Vondrak filter will have a good effect on separating low frequency signals from high frequency signals.

### 4.2. Genetic Algorithms

Genetic algorithms (GAs) behave as an adaptive search metaheuristic in natural evolution [21]. In a genetic algorithm, a population of individual solutions to an optimization problem is introduced toward a set of more optimal solutions. The evolution process usually starts from a sample set of potential individuals which is called population. In every iterative process, a new generation will be generated. The value of the objective function is computed in the optimization problem to evaluate the fitness of every individual in each generation. The more fit individuals from the current population are stochastically selected if it satisfies a specific selection criterion. At the same time, each individual's genome is developed to form a new generation by two operators: crossover and mutation. The new generation of candidate solutions is then input in the next iteration process of the algorithm. When the generation has produced to a maximum number, or the population has reached a satisfactory fitness level, the genetic algorithms will stop [22].

**Figure 4.**
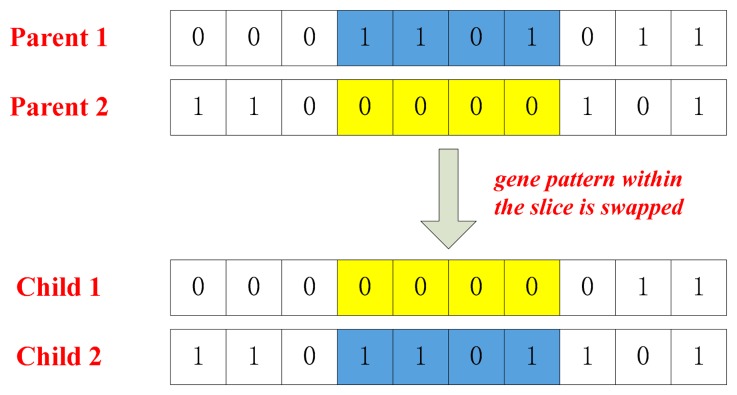
Crossover operation.

**Figure 5.**
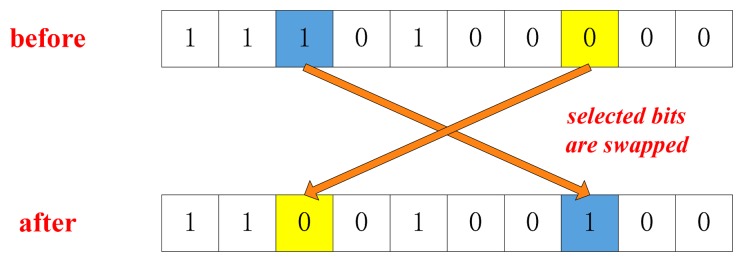
Mutation operation.

A typical genetic algorithm requires three most important aspects [23]: (1) the genetic representation of the solution domain; (2) the genetic operators of the solution domain; (3) an objective function to evaluate the solution domain. Steps of a general GA process are as follows:

(1) Initial: generate initial parent population and definite the crossover and mutation probability;
(2) Selection: evaluate the objective function and select chromosomes for reproduction;
(3) Crossover and Mutation: create offspring using reproduction operators such as crossover (Figure 4) and mutation (Figure 5);
(4) Termination: repeat the generational process until a termination condition has been reached.

The Vondrak filter is applied as low pass filter based on the frequency domain. The value of the smoothing factor is the key to low pass performance, so the objective function for the GA is constructed based on the frequency characteristic used to choose the smoothing factor. According to the above analysis of the frequency characteristics of force observation, the cut-off frequency of the low pass filter is 0.5 Hz. The objective conditions can be written as:

(25) $$\Delta_{L}=\text{mean}\left(S_{L}-S_{L}^{\prime }\right)\to 0$$

(26) $$\Delta_{H}=\text{mean}\left(S_{H}-S_{H}^{\prime }\right)\to \infty$$

where *S_L_* and *S*′*_L_* are the spectral analysis values of the force observation series in the low frequency domain from 0 Hz to 0.5 Hz before and after the Vondrak filter application, *S_H_* and *S*′*_H_* are the spectral analysis values of the force observation series in the high frequency domain from 0.5 Hz to the end before and after the Vondrak filter application.

When Δ*_L_* is tending to zero, the low frequency part of data is preserved after the Vondrak filter is used. At the same time, the high frequency part of the data is removed with Δ*_H_* trending to ∞. If the above two conditions are met, it indicates that the Vondrak filter is able to eliminate the high frequency noise and preserve the low frequency signal.

Based on Equations (25) and (26), the objective function used in the GA to choose the smoothing factor is constructed as:

(27) $$\varphi \left(\theta \right)=e^{\Delta_{L}}\cdot \frac{\ln\Delta_{H}+1}{\ln\Delta_{H}}$$

The spectral analysis results will be different as the smoothing factor is changing, so ϕ is the function with θ as the independent variable. When the function ϕ tends to one, the most appropriate smoothing factor will be chosen. In practical application, the threshold value which is used to determine whether the GA process ends is set to 0.995. If the function ϕ is larger than 0.995, the GA process is stopped and the smoothing factor is chosen.

## 5. The Proposed Method

**Figure 6.**
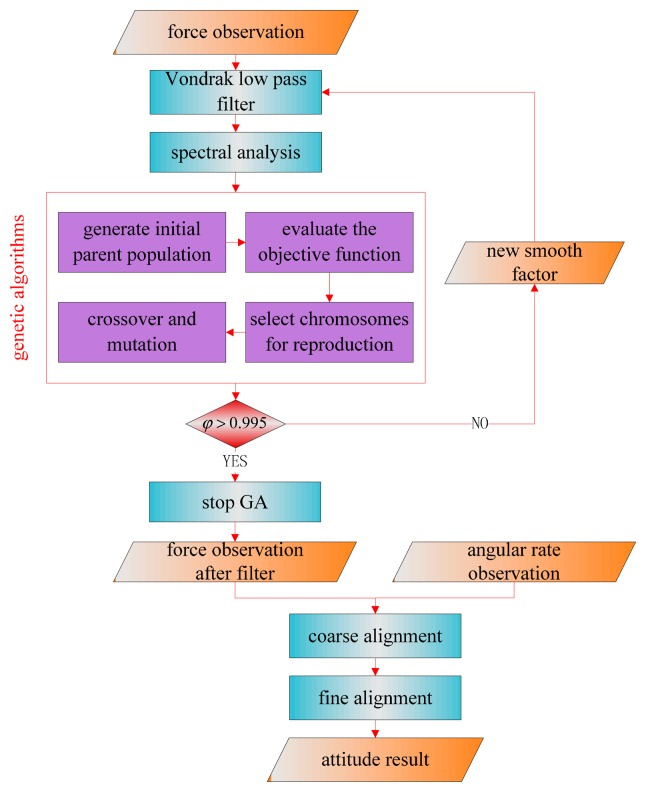
The improved initial alignment with a Vondrak filter for the disturbed base.

We schematically present a block diagram in Figure 6 to outline the fundamental mechanism of the improved initial alignment approach based on the Vondrak filter.

The Vondrak low pass filter is implemented for the force observations. A spectral analysis method is used to process the data after Vondrak filter application. The smoothing factor is chosen by the GA method. The parameters of genetic algorithms determined by experience are shown in the Table 1. In the GA method, the above parameters only affect the number of iterations and running time, but make little or no difference to the final result. The result of spectral analysis is one criterion in the process of parameter selection and Equation (27) is regarded as the objective function. If the function value is more than the threshold, the new smoothing factor will input to the Vondrak filter and the filter will repeat. Otherwise, the attitude matrix is calculated with the force observation after the filter and angular rate observation to get a more accurate mathematical platform for the initial alignment.

## 6. Results and Discussion

In order to test the proposed method based on the Vondrak filter, a variety of inertial navigation simulations are performed. The simulation conditions are a location at North latitude 37.6° and East longitude 108.6°. The simulations were conducted using two IMU systems with tactical grade and navigation grade sensors. The measurement parameters for each sensor are listed in Table 2. The gyroscope and accelerometer measurements are generated at a fixed frequency of 200 Hz. At the same time, a disturbance error is added in the simulation data. The disturbance error model is the sum of sine function with different frequencies. The error model is written as:

(28) e(t)=[sin(2πt)+sin(10πt)+sin(20πt)+sin(40πt)+sin(100πt)]×Bias/2

**Figure 7.**
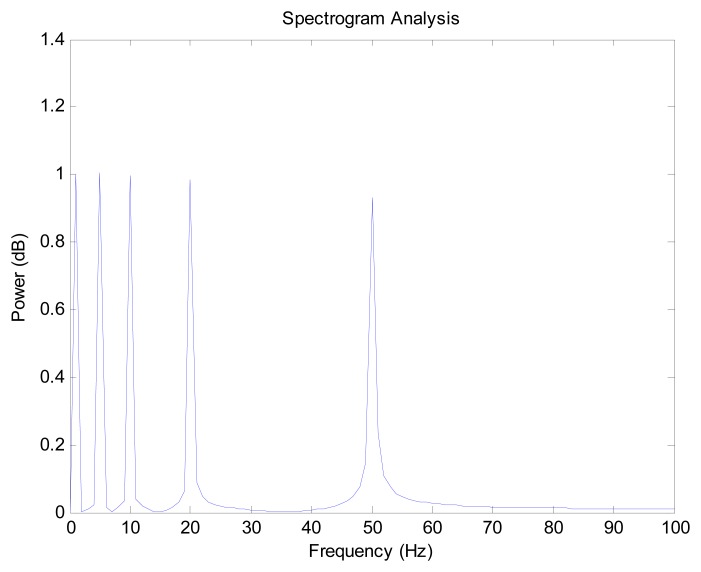
The spectrogram analysis of the error model.

Because the trigonometric function can be constructed with different frequencies, it is used to generate the disturbance noise. In the model, the main frequencies of the error model are 1, 5, 10, 20 and 50 Hz. The spectrogram analysis of the error model is shown in Figure 7.

**Figure 8.**
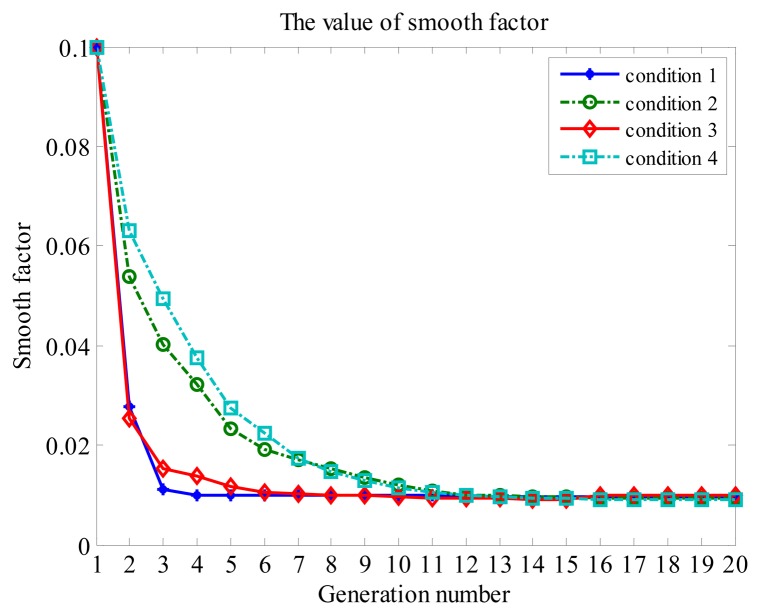
The value of the smoothing factor under different conditions.

The convergence process of the smoothing factor in GA for different conditions is shown in Figure 8. Because there is no disturbance in the observation, the convergence rate is very fast in Conditions 1 and 3. When the generation number is more than ten, the smooth factor converges to a stable value under Conditions 2 and 4.

**Figure 9.**
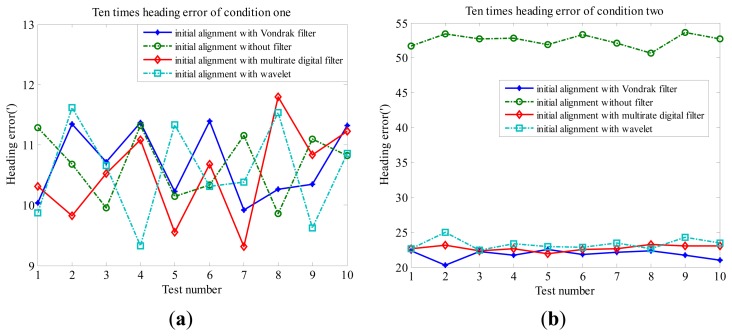

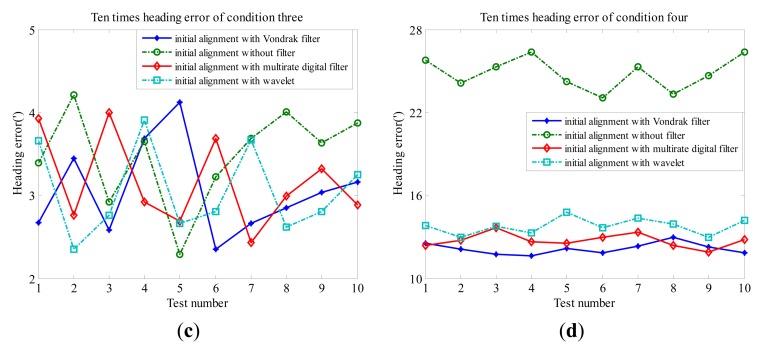
Ten times heading error of different schemes: (**a**) condition one; (**b**) condition two; (**c**) condition three; (**d**) condition four.

Figure 9 show the attitude errors of ten tests with different schemes. Except for Scheme 1 (initial alignment with the Vondrak filter) and Scheme 2 (initial alignment without filter), the other low-pass filter schemes are performed as a comparison to verify the performance of the proposed method: initial alignment with multirate digital filter [10] (Scheme 3) and initial alignment with wavelet [8] (Scheme 4). The heading error is chosen for analysis of the attitude error. Comparing different schemes, the alignment accuracy is almost as in the conditions of the observation without disturbance. It is noticed that the error of Scheme 1 is smaller than Scheme 2 under the circumstance that there is disturbance noise in the force observation. It is clear that the attitude accuracy has been greatly enhanced after the Vondrak filter is applied. The alignment is at least 2–3 times more accurate. Scheme 1, Scheme 3 and Scheme 4 are up to the nearly the same alignment grade in the conditions of the observation with disturbance. The performance of Scheme 1 is slightly better than that of Scheme 3 and Scheme 4.

**Figure 10.**
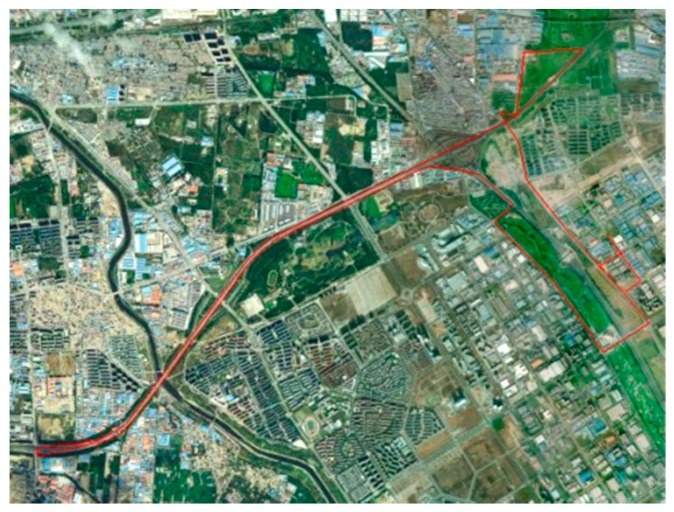
The test trajectory.

Field tests were also conducted to evaluate the performance of the proposed method. The test system is comprised of two sets of Leica GPS receivers and two IMU sets including a tactical grade IMU and navigation grade IMU. During the test, raw IMU data and GPS data were collected throughout the test navigation. One of the Leica receivers was set up as a reference station and the other one was used as a roving receiver with its antenna above the roof of the test vehicle. One Hz GPS data and 200 Hz INS data were received and stored in a notebook computer. Firstly, the vehicle is static for 10 min with outside disturbance to align the IMU. Then, the vehicle travels for half an hour. The kinematic navigation result is used to verify the alignment accuracy. The test trajectory is shown in Figure 10. Because the real initial attitude of the IMU is hard to acquire, the position accuracy using only INS with different alignment methods is computed to compare the alignment accuracy. The GPS observation was processed using the GPS software GrafNav™ 8.0 in DGPS mode and the solution was regarded as the position reference. The specifications of the IMU are given in Tables 3 and 4, respectively.

**Figure 11.**
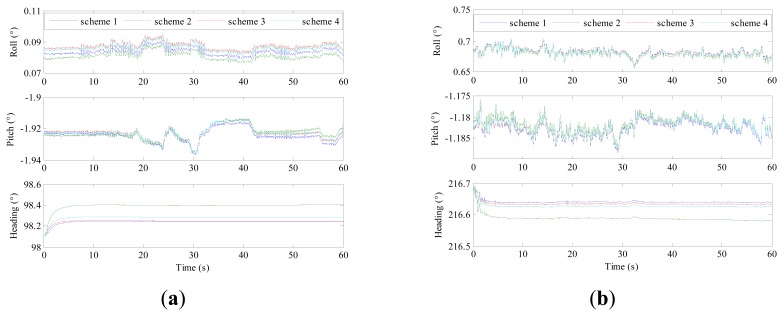
The fine alignment process results: (**a**) tactical grade IMU; (**b**) navigation grade IMU.

Figure 11 depicts the fine alignment results of the tactical grade IMU and navigation grade IMU by two schemes. The test condition is outdoors and some disturbance, such as wind, would be introduced, so the base is swaying slightly. The rate of convergence of alignment is faster in Scheme 1 than Scheme 2, and this is mainly reflected in the heading angle. The Vondrak filter is able to accelerate the alignment filter convergence. This suggests that the data after the Vondrak filter is used has higher accuracy. In the field test, the real attitude of an IMU is difficult to obtain, so it is difficult to definitely determine the alignment performance of different schemes according to Figure 10. More comparison is made in the following section.

After THE alignment process in THE disturbed base, the test vehicle with sensors would be driving in the city for half an hour. The position accuracy by INS itself will be computed based on the alignment results above. The resolution by DGPS mode is regarded as position reference.

**Figure 12.**
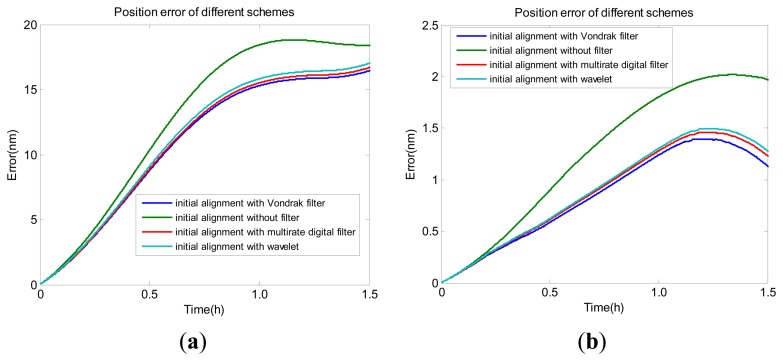
The navigation position error: (**a**) tactical grade IMU; (**b**) navigation grade IMU.

Shown in Figure 12 are time series of position errors of the tactical grade IMU and navigation grade IMU compared to the reference in different schemes.

Table 5 illustrates root mean square (RMS) and maximum value of the position errors. Both sets of figures show that the scheme based on the alignment method proposed in the paper provides the better navigation results.

Compared with Scheme 2, the position accuracy for the tactical grade and navigation grade IMUs are improved by 15.5% and 37.0%, respectively, for Scheme 1. The largest position errors for the tactical grade and navigation grade IMUs are 18.815 m and 2.018 m, when Scheme 2 is used. To the contrary, the largest position errors are 16.433 m and 1.396 m, respectively, when the proposed approach is applied. The navigation accuracy of Scheme 1 is superior to the accuracy of Scheme 3 and Scheme 4. Compared with Scheme 3 and Scheme 4, the alignment accuracy errors for the tactical grade IMU and navigation grade IMU drop to 12.100 nm and 0.954 nm, respectively, for Scheme 1. Because the raw data and computation method of INS self-navigation by itself is the same in the above four schemes, the accuracy of the initial attitude is the only one factor that influences the position navigation result. The better position navigation performance of Scheme 1 indicated that the improved initial alignment method proposed in the paper is able to achieve a more accurate attitude than the conventional method without filter. At the same time, the performance of the Vondrak filter is a little better than that of the other low-pass filters, such as digital filter and wavelet. Another important point here is that the digital filter and wavelet need to set more parameters than the Vondrak filter. On the contrary, the smoothing factor in the Vondrak filter can be computed by the GA method, so there are no other parameters which need to be set, besides the cut-off frequency.

## 7. Conclusions

This paper proposes a novel initial alignment with a Vondrak low pass filter to improve the alignment accuracy for a disturbed base. The Vondrak low pass filter is constructed to reduce the high frequency noise in force observations. In addition, the genetic algorithms method is implemented to choose the smoothing factor in the Vondrak filter and the corresponding objective condition is built. Simulated and real measurements were used to demonstrate the performance of the proposed approach.

The spectral analysis shows that there is high frequency noise in force observations under disturbed base circumstances. The simulation and measured data results indicate that the proposed initial alignment method can provide a slightly better performance for a disturbed base IMU than the other low-pass filters. After the alignment method proposed in the paper, the position error of INS alone navigation is less than that of the conventional method without filter. Compared with the other filter methods, the Vondrak low pass filter only uses the cut-off frequency parameter to realize the low-pass filter. The smoothing factor in the Vondrak filter can be computed with the cut-off frequency and optimized by the GA method. Hence, the proposed method is easier to implement than other low-pass filters. The proposed filter method is mainly used to remove the high frequency noise. In the paper, a vehicle with wind disturbance is regarded as the research object and the performance is good. In addition, the other high frequency noise, such as the engine disturbance for aircraft, plasma oscillation and so on, can also be eliminated by the proposed method. For our future work, the potential of the Vondrak filter for other environmental conditions with multi-frequency noise, such as in a sailing ship, should be investigated.

## Figures and Tables

**Table 1.** Parameters of genetic algorithms.

**Parameters**	**Value**
Crossover Probability	70%
Mutation probability	5%
Population size	20

**Table 2.** Simulation technical data in different conditions.

**Condition**	**Grade**	**Gyro**		**Accelerometer**		**Disturbance**

		**Drift Rate**	**Gaussian Noise**	**Bias**	**Gaussian Noise**	
1	Tactical	0.1°/h	N(0, (0.05 deg/h)^2^)	5 × 10^−5^ g	N(0, (5 × 10^−5^ g)^2^)	NO
2	Tactical	0.1°/h	N(0, (0.05 deg/h)^2^)	5 × 10^−5^ g	N(0, (5 × 10^−5^ g)^2^)	YES
3	Navigation	0.02°/h	N(0, (0.01 deg/h)^2^)	1 × 10^−5^ g	N(0, (2 × 10^−5^ g)^2^)	NO
4	Navigation	0.02°/h	N(0, (0.01 deg/h)^2^)	1 × 10^−5^ g	N(0, (2 × 10^−5^ g)^2^)	YES

**Table 3.** Tactical grade IMU technical data.

**Parameters**	**Gyro**	**Accelerometer**
Bias	0.1 deg/h	5 × 10^−5^ g
Scale factor	100 ppm	100 ppm
Random walk	0.05 deg/h/sqrt (Hz)	5 × 10^−5^ g/sqrt (Hz)

**Table 4.** Navigation grade IMU technical data.

**Parameters**	**Gyro**	**Accelerometer**
Bias	0.02 deg/h	1 × 10^−5^ g
Scale factor	20 ppm	40 ppm
Random walk	0.01 deg/h/sqrt (Hz)	1 × 10^−5^ g/sqrt (Hz)

**Table 5.** The navigation position error for different schemes.

**Scheme**	**RMS (nm)**		**MAX (nm)**	

	**Tactical Grade**	**Navigation Grade**	**Tactical Grade**	**Navigation Grade**
1	12.100	0.954	16.433	1.396
2	14.326	1.514	18.815	2.018
3	12.268	1.003	16.672	1.460
4	12.522	1.029	17.007	1.497
